# Predicting intraoperative hemorrhage during curettage treatment of cesarean scar pregnancy using free-breathing GRASP DCE-MRI

**DOI:** 10.1186/s12884-023-06188-y

**Published:** 2024-01-03

**Authors:** Zhi-Gang Wang, Feng-Leng Yang, Chun-Ying Liu, Fang Wang, Ying Xiong, Qiang Zhang, Mei-ning Chen, Hua Lai

**Affiliations:** 1 Department of Radiology, Chengdu Women’s and Children’s Central Hospital, School of Medicine, University of Electronic Science and Technology of China, No.1617 of Riyue Avenue, Qingyang District, Chengdu, 610091 China; 2 Department of Gynecology, Chengdu Women’s and Children’s Central Hospital, School of Medicine, University of Electronic Science and Technology of China, Chengdu, China; 3 Department of MR Scientific Marketing, Siemens Healthineers, Shanghai, China

**Keywords:** Cesarean scar pregnancy, Dynamic magnetic resonance imaging, Dynamic contrast enhancement, Prediction ability, Surgical risk

## Abstract

**Objective:**

To explore the feasibility of the golden-angle radial sparse parallel (GRASP) dynamic magnetic resonance imaging (MRI) technique in predicting the intraoperative bleeding risk of scar pregnancy.

**Methods:**

A total of 49 patients with cesarean scar pregnancy (CSP) who underwent curettage and GRASP-MRI imaging were retrospectively selected between January 2021 and July 2022. The pharmacokinetic parameters, including Wash-in, Wash-out, time to peck (TTP), initial area under the curve (iAUC), the transfer rate constant (Ktrans), constant flow rate (Kep), and volume of extracellular space (Ve), were calculated. The amount of intraoperative bleeding was recorded by a gynecologist who performed surgery, after which patients were divided into non-hemorrhage (blood loss ≤ 200 mL) and hemorrhage (blood loss > 200 mL) groups. The measured pharmacokinetic parameters were statistically compared using the t-test or Mann–Whitney U test with a significant level set to be *p* < 0.05. The receiver operating characteristic (ROC) curve was constructed, and the area under the curve (AUC) was calculated to evaluate each parameter’s capability in intraoperative hemorrhage subgroup classification.

**Results:**

Twenty patients had intraoperative hemorrhage (blood loss > 200 mL) during curettage. The hemorrhage group had larger Wash-in, iAUC, Ktrans, Ve, and shorter TTP than the non-hemorrhage group (all *P* > 0.05). Wash-in had the highest AUC value (0.90), while Ktrans had the lowest value (0.67). Wash-out and Kep were not significantly different between the two groups.

**Conclusion:**

GRASP DCE-MRI has the potential to forecast intraoperative hemorrhage during curettage treatment of CSP, with Wash-in exhibiting the highest predictive performance. This data holds promise for advancing personalized treatment. However, further study is required to compare its effectiveness with other risk factors identified through anatomical MRI and ultrasound.

## Introduction

Cesarean scar pregnancy (CSP) is a rare form of ectopic pregnancy with potentially severe complications such as uterine rupture and massive bleeding, which are life-threatening [1]. Its incidence has been reported as approximately 1:2,000 pregnancies and has been increasing in recent years [2]. Once diagnosed, the immediate termination of pregnancy is required, commonly achieved by surgery, i.e., suction curettage [3]. However, uncontrollable intraoperative hemorrhage may appear during treatment, resulting in a hysterectomy and loss of future fertility. Therefore, preoperative prediction of the potential risk of major intraoperative bleeding is crucial for clinical practice. Recent studies have shown that pregnancy age, gestational age, serum human chorionic gonadotropin (b-hCG), gestational sac (GS), CSP mass size (≥ 6 cm), myometrial layer thickness, menolipsis time, and peritrophoblastic perfusion are independent risk factors for excessive intraoperative hemorrhage [3, 4]. Yet, most patients with CSP show no specific symptoms, and diagnosis depends on recognizing the GS at the uterine incision, which can be defied using MRI or ultrasound [5].

The value of MRI in diagnosing CSP has been gradually recognized [6]. Studies have shown that MRI is superior to ultrasound because MRI can clearly show the position of the GS in relation to the incisional scar morphologically and clarify whether the incisional scar is adhering to the surrounding muscular layer, whether the GS sac is in bed, and whether there is blood accumulation around the uterine cavity [7]. Over the years, dynamic contrast-enhanced magnetic resonance imaging (DCE-MRI) has been used to locate GS and assess neovascular perfusion [8]. However, conventional DCE-MRI is sensitive to motion and requires breath-holding during a scan, resulting in poor temporal and spatial resolution. To overcome this limitation, a novel DCE-MRI technique known as golden-angle radial sparse parallel (GRASP) imaging was invented to achieve a much higher temporal resolution without breath-holding [7], thus increasing patient comfort while allowing higher spatial resolution images to be acquired and more accurate perfusion to be quantified. GRASP has been widely used in various parts of the body, including the liver, prostate, breast, bladder, and kidney [9–14]. The free-breathing GRASP technique is motion-insensitive and enables acquiring images with high temporal and spatial resolution. The high spatial resolution images further enable anatomical-like observation of the lesion, while the high temporal resolution images improve the accuracy of quantitative perfusion parameters [15]. However, to our knowledge, no study has applied GRASP to the diagnostic imaging of CSP.

This study aimed to explore the feasibility of using GRASP DCE-MRI to characterize peritrophoblastic perfusion and evaluate its prediction power for intraoperative hemorrhage during curettage of CSP.

## Materials and methods

### Patients

A total of 49 patients with CSP who underwent curettage and received no previous interventions were retrospectively selected between January 2021 and July 2022. Inclusion criteria were the following: (1) diagnostic criteria for incisional scar pregnancy were met: history of cesarean delivery; elevated serum HCG levels; Doppler examination reveals an empty uterine cavity and cervical canal, the GS in the anterior isthmic area of the uterus, a thin or absence layer of myometrium between the GS and the bladder, and circular blood flow around the GS [16]; (2) a voluntary termination of pregnancy; (3) termination of pregnancy by surgical method; (4) postoperative pathology confirmed a CSP; (5) MRI scan performed 3 days before surgery and GRASP dynamic enhancement technique was used. Exclusion criteria were: (1) twin pregnancy; (2) those who have received medical treatment before termination of pregnancy; (3) incomplete clinical data; (4) poor MRI image quality. This study was approved by the medical ethics committee of Chengdu women’s and children's Central Hospital (Approval number: 2021 (107)), and the written informed consent of the research subjects was also obtained.

### MRI

All MRI scans were acquired using a 3.0 T MR system (MAGNETOM Vida, Siemens Healthineers) with an 18-channel body phased array surface coil. The patient’s bladder was moderately full. The patient was in the supine position, and the uterus was scanned on transverse axis T2WI, T2WI fat suppression sequence and DWI, sagittal T1WI, T2WI, and T2WI fat suppression sequence and coronal T2WI. For dynamic contrast-enhanced scanning, gadopentetate dimeglumine (Gd-DTPA) was intravenously injected at a dose of 0.1 mmol/kg and a flow rate of 3 ml/s. After a 15-s delay, GRASP acquisition was performed using the following parameters: matrix = 320 × 320, FOV = 256 mm × 256 mm, slice thickness = 4 mm, TR = 4.09 ms, TE = 2.04 ms, temporal resolution = 10.4 s, and phases = 25. 2.3 Image processing.

**Fig. 1:**
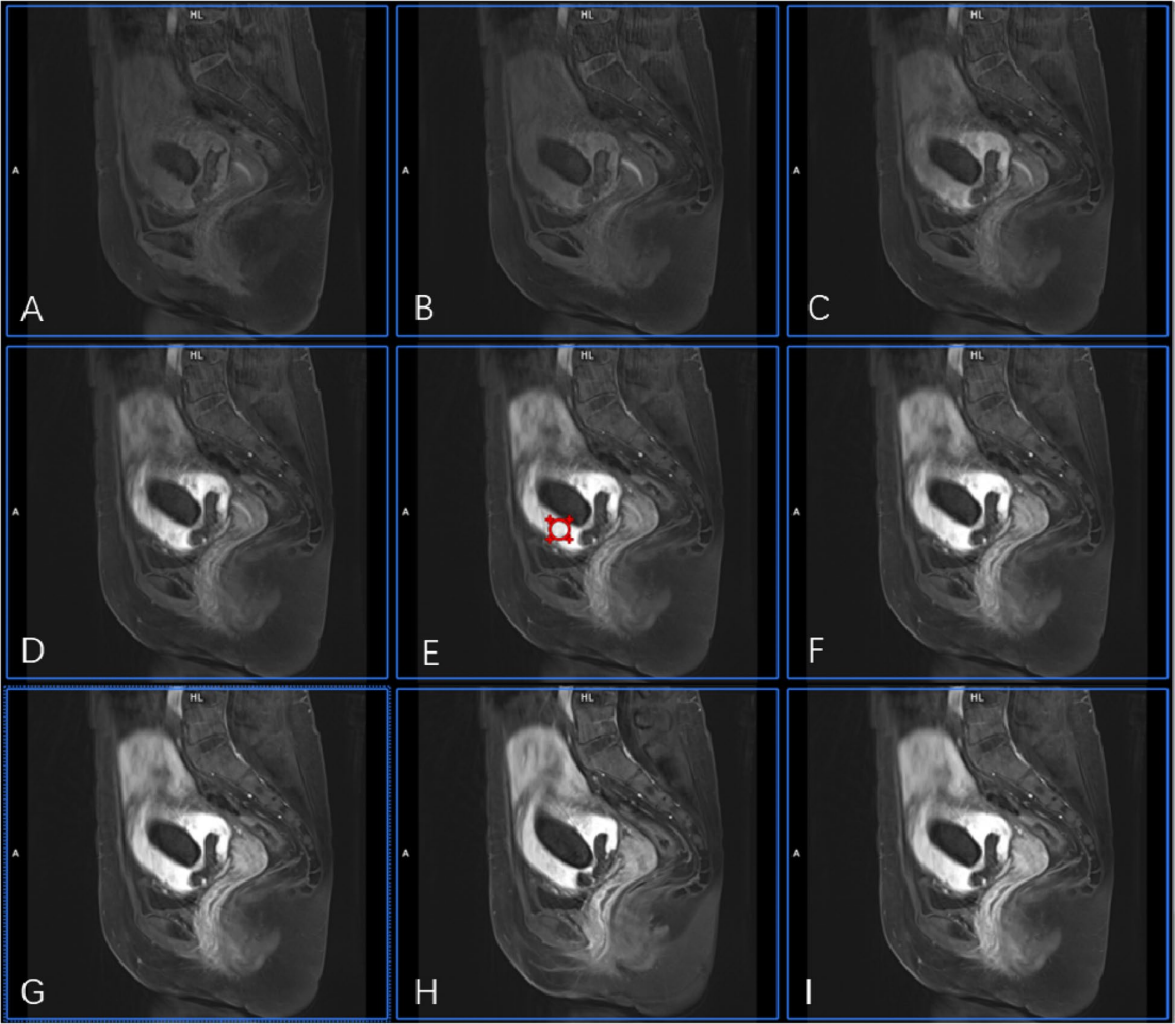
Drawing ROI on strongest contrast-enhancing GRASP image. A 32-year-old woman with CSP 56 days after the cessation of menses. **A-I** shows a dynamic contrast-enhanced phase from 3 to 11 in the GRASP image. GS villi around the first begin to enhance, and the ROI was drawn on the strongest contrast-enhancing region

**Fig. 2:**
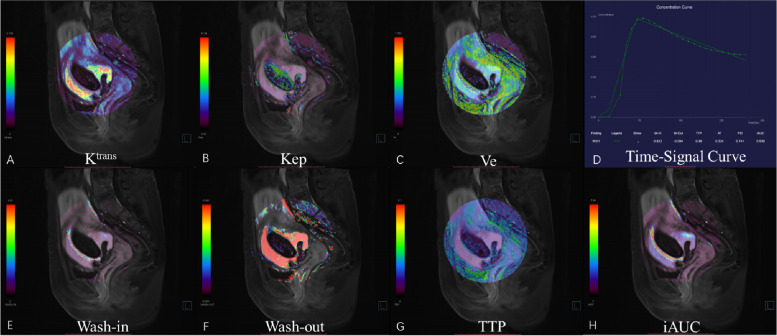
GRASP pharmacokinetic images in a 32-year-old woman (menopause time: 56 days) with hemorrhage (blood loss > 200 mL) during curettage. **A-H** shows the time-signal curve and pharmacokinetic parameters, including Wash-in, Wash-out, TTP, iAUC, K^trans^, Kep, and Ve, respectively. A high signal around the GS villi was observed in Wash-in, iAUC, K^trans^, Ve, and a low signal in TTP

**Fig. 3:**
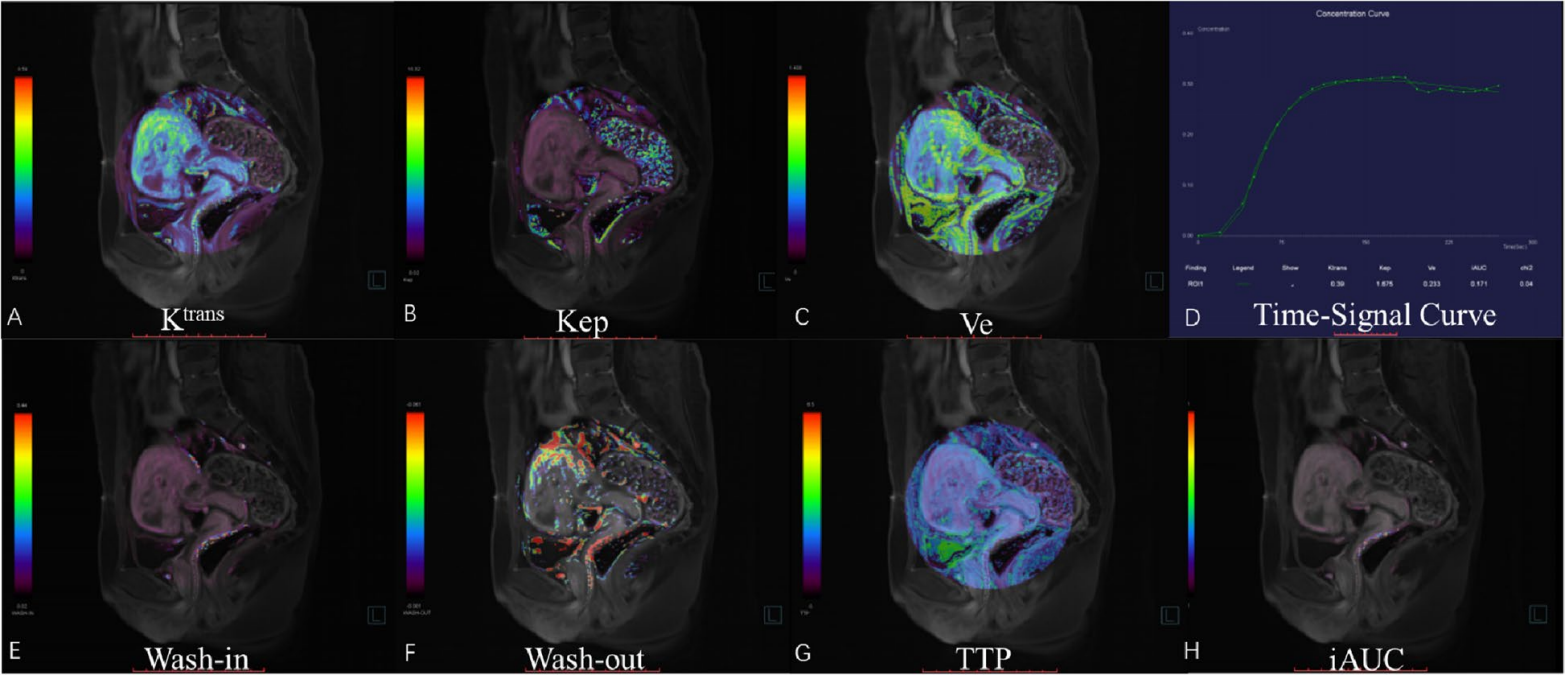
GRASP pharmacokinetic images in a 24-year-old woman (menopause time: 44 days) with non-hemorrhage (blood loss ≤ 200 mL) during curettage. **A-H** shows the time-signal curve and pharmacokinetic parameters, including Wash-in, Wash-out, TTP, iAUC, K^trans^, Kep, and Ve, respectively. Low signal around the GS villi was observed in Wash-in, iAUC, K^trans^, Ve, and high in TTP

The images were analyzed by Tissue 4D (Siemens Healthcare, Erlangen, Germany). First, images from the phase with the strongest contrast-enhancing were selected, and the region of interest (ROIs) was defined on enhanced GS villi around the previous incision scar (Fig. 1). This was done independently by two radiologists (Dr. Wang, with 15 years of experience, and Dr. Lai, with 20 years of experience) who were blinded to the degree of hemorrhage during curettage. Then, pharmacokinetic parameters, including Wash-in, Wash-out, time to peck (TTP), iAUC, Ktrans, Kep, and Ve were calculated within the ROIs and averaged between the two raters (Figs. 1, 2 and 3).

### Data collection

Two physicians with deputy senior professional titles blindly analyzed the films to select the villi in the incision area and delineate the ROI and recorded the semi-quantitative and quantitative analysis values (Wash-in, Wash-out, TTP, Ktrans, Kep, and iAUC). The quantitative index uses the average of the two measurements.

### Clinical record

Two gynecologists with over 10 years of operation experience completed the uterine cavity curettage and recorded the postoperative bleeding volume in the medical records. The amount of intraoperative bleeding was recorded by a gynecologist who performed a curettage. Then, patients were divided into two groups: non-hemorrhage (blood loss ≤ 200 mL) and hemorrhage (blood loss > 200 mL) [17].

### Statistical analysis

Analysis was conducted using SPSS (version 22.0). To assess the statistical consistency of the measurement data obtained from two doctors, the intra-group correlation coefficient was employed. A coefficient of less than 0.5 was indicative of poor consistency, while values in the range of 0.5 to 0.7 signified moderate consistency. Coefficients falling between 0.7 and 0.9 suggested relative consistency, and values exceeding 0.9 indicated excellent consistency. Continuous variables following a normal distribution were summarized as mean ± standard deviation, whereas skewed distributed variables were presented as medians along with their upper and lower quartiles. To compare differences between groups for these two types of variables, unpaired t-tests were utilized for normally distributed variables, and the non-parametric equivalent, the Mann-Whitney U test, was employed for skewed distributed variables. For the prediction of major bleeding, ROC (Receiver Operating Characteristic) curve analysis was performed for each parameter that exhibited significant differences between groups. Cutoff values were determined by identifying the largest Youden Index (Youden index = sensitivity + specificity - 1). For each selected cutoff, positive predictive value, negative predictive value, and accuracy were calculated. A p-value of less than 0.05 was considered statistically significant in this analysis.

## Results

### Patients’ clinical data

A total of 49 patients with CSP [age range: 25–48 years, mean: 33.6 ± 3.9 years; intraoperative blood loss: 100 (20, 200) ml; number of previous cesarean sections: 1 (1, 2); menopause time: 42–90 days, mean: 55.41 ± 11.75 days; interval between current and last cesarean section: 1–8 years, mean: 4.06 ± 1.62 years; cases of virginal bleeding: 47/49] were retrospectively selected. There were 33 cases with vaginal bleeding, 14 with vaginal bleeding and lower abdominal pain, and 2 without obvious symptoms. Twenty had intraoperative hemorrhage (blood loss > 200 mL) during curettage. The consistency of the measured data of the two doctors was greater than 0.7, suggesting good consistency (Table 1).

**Table 1:** Consistency of measurement data of doctors A and B

	**Wash-in**	**Wash-out**	**TTP**	**iAUC**	**Ktrans**	**Kep**	**Ve**
**Dr. A**	0.52 ± 0.23	-0.03 ± 0.05	1.16 ± 0.29	0.31 (0.21, 0.45)	0.95 (0.30, 1.55)	2.71 (1.24, 3.96)	0.31 (0.25, 0.43)
**Dr. B**	0.53 ± 0.23	-0.03 ± 0.07	1.16 ± 0.24	0.38 (0.23, 0.57)	0.80 (0.43, 1.32)	2.70 (1.21, 3.49)	0.39 (0.25, 0.51)
**ICC**	0.789	0.768	0.778	0.928	0.906	0.910	0.705
**95% CI**	0.381–0.803	0.589–0.869	0.606–0.875	0.873–0.959	0.833–0.947	0.840–0.949	0.477–0.833

*ICC* interclass correlation coefficient, *95% CI* 95% confidence interval

Dr. A, Dr. Wang; Dr. B, Dr, Lai

### Correlation analysis of grasp semi-quantitative parameters in hemorrhage

The difference in wash-in, TTP, and iAUC indexes was statistically significant between patients with intraoperative hemorrhage (blood loss > 200 mL) during curettage and those without hemorrhage (all *P* < 0.05), while there was no difference in wash-out value (*P* > 0.05) (Table 2).

**Table 2:** Correlation analysis of GRASP semi-quantitative parameters in hemorrhage

	** < *200 ml***	** ≥ *200 ml***	***T/Z***	***P***
**Wash-in**	0.41 ± 0.14	0.69 ± 0.17	*T* = -6.276	0.000
**Wash-out**	-0.03 ± 0.05	-0.04 ± 0.07	*T* = 0.995	0.325
**TTP (s)**	1.28 ± 0.21	0.98 ± 0.17	*T* = 5.329	0.000
**iAUC**	0.25 (0.17, 0.37)	0.52 (0.38, 0.75)	*Z* = -3.885	0.000

*TTP* time to peak

### Correlation analysis of grasp quantitative parameters in hemorrhage

The difference in Kep index was not statistically significant (*P* > 0.05) between patients with intraoperative hemorrhage (blood loss > 200 mL) during curettage and those without hemorrhage (all *P* < 0.05), while Ktrans and Ve were statistically significant (all *P* < 0.05) (Table 3).

**Table 3:** Correlation analysis of GRASP quantitative parameters in hemorrhage

	** < *200 ml***	** ≥ *200 ml***	**Z**	***P***
**Ktrans (min**^**−1**^**)**	0.72 (0.34, 1.16)	1.16 (0.57, 1.92)	Z = -1.973	0.048
**Kep (min**^**−1**^**)**	2.41 (1.35, 3.61)	3.11 (1.00, 4.59)	Z = -0.712	0.476
**Ve**	0.29 (0.25, 0.36)	0.43 (0.37, 0.58)	Z = -3.885	0.000

### GRASP index and efficacy of bleeding risk prediction

The results of four positive indicators, including Wash-in, TTP, iAUC, Ktrans, and Ve were analyzed according to the ROC curve. The AUC values of Ktrans and Ve indexes in predicting major intraoperative bleeding were 0.667 (95%CI: 0.501 ~ 0.833) and 0.829 (95%CI: 0.707 ~ 0.952), with the optimal cutoff values of Ktrans and Ve being 0.983 and 0.368, respectively. The AUC values of Wash-in, TTP, and iAUC for predicting major intraoperative bleeding were 0.903 (95%CI: 0.818 ~ 0.988), 0.878 (95%CI: 0.779 ~ 0.976), and 0.884 (95%CI: 0.793 ~ 0.975), with the optimal cutoff values of Wash-in, TTP, and iAUC being 0.548, 1.12, and 0.374, respectively. TTP had the highest sensitivity, specificity, positive predictive value, and negative predictive value (Table 4).

**Table 4:** GRASP index and efficacy of bleeding risk prediction

	**AUC**	**Youden index**	**The optimal cutoff value**	**Sensitivity (%)**	**Specificity (%)**	**Positive predictive value (%)**	**Negative predictive value (%)**
**Ktrans (min**^**−1**^**)**	0.667	0.336	0.983	70	58.6	53.8	73.9
**Ve (min**^**−1**^**)**	0.829	0.593	0.368	80	79.3	72.7	85.2
**Wash-in**	0.903	0.678	0.548	85	82.8	77.2	88.9
**TTP (s)**	0.878	0.712	1.121	85	86.2	81.0	89.3
**iAUC**	0.884	0.628	0.374	80	82.8	76.2	85.7

*TTP* time to peak

## Discussion

To our knowledge, this is the first study to report on GRASP DCE-MRI's feasibility for predicting intraoperative hemorrhage during surgical treatment of CSP. Histologically, the depth of trophoblast invasion has become a common proxy for the classification of CSP. Placentation extravillous trophoblasts modify uterine vessels to promote placental blood flow, and cytotrophoblasts give rise to placental villi that undergo vasculogenesis and angiogenesis [18]. This, in turn, leads to peritrophoblastic hyperperfusion but at a variable degree in patients with CSP, which was demonstrated by our study's perfusion quantified by GRASP DCE-MRI. According to their pharmacokinetic properties, the larger Wash in, iAUC, and shorter TTP values reflected increased blood supply while the larger Ktrans and Ve reflected increased vessel permeability, and this probably explains why patients with these kinds of perfusion could be reliably assigned to the hemorrhage group. With this perfusion quantification capability, plus its excellent anatomical contrast for the GS and myometrial layer, MRI is anticipated to be a better comprehensive intraoperative hemorrhage risk assessment tool for CSP than ultrasound.

GRASP-MRI technology can be customized to flexibly reconstruct at any time resolution, capture characteristic enhancement, improve the accuracy of differential diagnosis and the quantitative accuracy of quantitative perfusion analysis, and obtain morphological enhancement information and quantitative analysis results with one scan [19, 20]. In addition, it has a better high spatial resolution, improves the sensitivity of detecting small lesions, and makes the relationship with surrounding tissues clear [21]. The scanning time of traditional pelvic dynamic enhancement in 8 stages is about 2 min and 38 s, and the scanning time of GRASP-MRI technology is about 4 min and 41 s. Although the time is slightly increased, 25 stages of dynamic images can be obtained, which can provide accurate time dynamic curve images, improve the judgment of the nature of the lesions, and obtain more precise hemodynamic information without increasing the complexity of the scanning process [22].

The quantitative and semi-quantitative parameters of GRASP are permeability parameters derived from complex pharmacokinetic models, which include Wash-in, Wash-out, TTP, iAUC, Ktrans, Kep, and Ve. This study analyzed the risk of intraoperative bleeding in CSP by semi-quantitative and quantitative parameters. Our data suggests that among the significant parameters for discrimination, Wash-in exhibits the highest diagnostic performance (AUC: 0.903) compared to the others. We also employed the Youden index to determine the optimal cut-off value for each parameter. Remarkably, we observed that the TTPs at the cut-off values with the highest Youden index exhibited an excellent balance between sensitivity and specificity. Although this study was conducted with a limited sample size, introducing a degree of uncertainty, it implies that MRI parameters with appropriate values can prove valuable for both ruling in and ruling out clinical scenarios.

Wash-in refers to the filling time of the contrast agent in the tissue, reflecting the degree of early perfusion. The increase in Wash-in indicates an increase in blood perfusion in the region. Usually, the villi at the implantation site are more extensive and active, so the blood supply is abundant.

TTP refers to the time when tissue enhancement reaches its peak. It can reflect the permeability of blood vessels [23]. If the TTP value is low, the contrast agent enters the extravascular space quickly through the vascular endothelial cells, and the blood flow is large, which can easily cause massive bleeding.

iAUC refers to the area under the time concentration curve within 1 min after contrast agent injection, which is related to the tissue blood supply and surrounding space and represents the rate and total amount of contrast agent entering the tissue [24]. The large Wash-in and iAUC values and the short TTP values reflect the increase in tissue cell function and regional blood perfusion, which increase the risk of bleeding.

Ktrans reflects the rate constant of the contrast agent entering the extracellular space from the vascular lumen, and Ve represents the volume fraction of the extracellular space of the microvascular tissue. It is an important index to reflect the degree of cell differentiation, vascular surface permeability, and vascular cell integrity [24].

Overexpression of vascular endothelial growth factor and cytokines released by uterine natural killer cells enhances the invasiveness of extravillous trophoblasts cells and increases vascular permeability. If there is more blood flow at the implantation site, the higher the Ktrans and Ves value, the more likely it is to cause massive bleeding [25]. This study found that the Ktrans and Ve values can predict the risk of bleeding, but the sensitivity, specificity, positive predictive value, and negative predictive value are lower than the wash-in, TTP, and quantitative iAUC index. It is considered that it may be related to sample selection. In some patients, the invasion of extravillous trophoblasts cells causes insignificant expression of vascular permeability due to early pregnancy. Some studies have suggested amenorrhea days (51 days) is the best critical value influencing bleeding factors during CSP [26]. In addition, the decrease in time resolution will also lead to the underestimation of Ktrans [27].

There was no statistically significant relationship between Wash-out and Kep parameters and major bleeding. Therefore, it is considered that GD-DTPA is the contrast agent in the extracellular space. It takes about 60-70 min for the concentration in serum to decrease by half. The enhancement mode of villus tissue can be divided into ascending platform type and slowly descending type. The contrast agent stays in the villi for a long time and enters the vascular lumen slowly from the extravascular space. The pattern of villus enhancement was inconsistent with that observed by some studies [8, 28]. It is hoped that the sample size can be expanded for further research.

This study presents several limitations. Firstly, it is a retrospective, pilot study with a small sample size, in which the findings need to be confirmed by future large-sample, multi-center studies. Secondly, the majority of high-risk patients had received interventional or mifepristone treatment prior to the operation, which could be a potential confounding factor. In addition, ROI was selected as the villus in the incision area. Whether this area is clearly the implantation site needs to be further explored. Finally, certain crucial factors, such as the size of the gestational sac, the thickness of the uterine scar, and the gestational weeks, were not taken into account. Consequently, additional research is warranted to investigate the impact of these factors on intraoperative bleeding in CSP.

## Conclusions

The GRASP DCE-MRI has the potential to forecast intraoperative hemorrhage during curettage treatment of CSP, thereby facilitating the selection of personalized treatment strategies. Future research is necessary to assess its efficacy in comparison with other risk factors identified through anatomical MRI and ultrasound.

## Data Availability

The datasets generated and/or analyzed during the current study are not publicly available because they contain the patients’ personal information but are available from the corresponding author upon reasonable request.
